# A hierarchical method to automatically encode Chinese diagnoses through semantic similarity estimation

**DOI:** 10.1186/s12911-016-0269-4

**Published:** 2016-03-03

**Authors:** Wenxin Ning, Ming Yu, Runtong Zhang

**Affiliations:** Health Care Services Research Center, Department of Industrial Engineering, Tsinghua University, Beijing, 100084 PR China; Department of Information Management, School of Economics and Management, Beijing Jiaotong University, Beijing, 100084 PR China

**Keywords:** Automated code assignment, Semantic similarity, Distributional semantics, Context vectors, Chinese diagnoses, ICD

## Abstract

**Background:**

The accumulation of medical documents in China has rapidly increased in the past years. We focus on developing a method that automatically performs ICD-10 code assignment to Chinese diagnoses from the electronic medical records to support the medical coding process in Chinese hospitals.

**Methods:**

We propose two encoding methods: one that directly determines the desired code (flat method), and one that hierarchically determines the most suitable code until the desired code is obtained (hierarchical method). Both methods are based on instances from the standard diagnostic library, a gold standard dataset in China. For the first time, semantic similarity estimation between Chinese words are applied in the biomedical domain with the successful implementation of knowledge-based and distributional approaches. Characteristics of the Chinese language are considered in implementing distributional semantics. We test our methods against 16,330 coding instances from our partner hospital.

**Results:**

The hierarchical method outperforms the flat method in terms of accuracy and time complexity. Representing distributional semantics using Chinese characters can achieve comparable performance to the use of Chinese words. The diagnoses in the test set can be encoded automatically with micro-averaged precision of 92.57 %, recall of 89.63 %, and F-score of 91.08 %. A sharp decrease in encoding performance is observed without semantic similarity estimation.

**Conclusion:**

The hierarchical nature of ICD-10 codes can enhance the performance of the automated code assignment. Semantic similarity estimation is demonstrated indispensable in dealing with Chinese medical text. The proposed method can greatly reduce the workload and improve the efficiency of the code assignment process in Chinese hospitals.

**Electronic supplementary material:**

The online version of this article (doi:10.1186/s12911-016-0269-4) contains supplementary material, which is available to authorized users.

## Background

The electronic medical record (EMR) is a rich source of clinical information for medical study and other applications related to quality of healthcare, clinical decision support, and reliable information flow among individuals and departments involved in patient care [1]. However, much of the information cannot be fully utilized because it is in free-text form, which increases the difficulty of searching, summarization, and statistical analysis [2]. A general approach is to map unstructured clinical texts to concepts within a standardized coding and classification system, such as the International Classification of Diseases (ICD), the latest version of which is ICD-10. The use of ICD codes has expanded from classifying morbidity and mortality statistics to diverse sets of applications, including reimbursement, administration, epidemiology, and health service research [3].

ICD-10 is the most widely used taxonomy of diagnosis codes in the world [4]. Each code describes a certain kind of disease, injury, or cause of death. All codes are organized hierarchically in a tree structure, where a child code represents a subdivision of its parent. For example, the category (3-digit) code A00 pertains to the condition “Cholera” and its subcategory (4-digit) code A00.0 pertains to the specific condition “Cholera due to Vibrio cholerae 01, biovar cholerae”. The section code A00-A09 subsumes a broader range of “Intestinal infectious diseases”. Codes at the section level or higher are generally not used as reference codes [5]. In practice, code assignment is usually performed at the subcategory level, which contains over 10,000 unique ICD-10 codes.

The code assignment process is manually implemented and requires trained staff with a good knowledge of coding rules and medical terminologies; hence, manual coding is rather time-consuming [6]. A method that can automatically assign ICD codes to clinical text would significantly enhance the efficiency of the medical coding process, especially in China where the adoption of hospital information systems and the accumulation of medical documents have rapidly increased in the past years [7]. A few approaches have been developed for the automated encoding of English medical text or documents, but very limited studies have focused on Chinese text. Existing methods of automated code assignment can generally be divided into two types. The first type is performed through the adoption or adaptation of existing medical language processing (MLP) tools that perform concept identification on a given document. The second type models the code assignment problem as a text classification problem (where each code is considered as a class label) and applies machine learning approaches to build classifiers for code assigning. Friedman et al. [8] introduced an effective method that adapts MedLEE, an existing MLP system, to extract clinical concepts from discharge summaries and map them to UMLS codes according to an automatically created encoding table. Kukafka et al. [9] and Lussier et al. [10, 11] also adopted MedLEE to encode discharge summaries under coding schemes ICF, ICD-9, and SNOMED. Besides, MTI [12] and MetaMap [13] also demonstrated promising performance in clinical concept extraction. However, MLP methods are limited because their encoding accuracy is largely dependent on the performance of MLP tools and the quality of the knowledge base. Machine learning methods can be utilized as an alternative. Pakhomov et al. [14] introduced an automated diagnosis encoding system implemented at the Mayo Clinic that combines a simple example-based method and the naïve Bayes algorithm. Yang and Chute [15] introduced an Expert Network for clinical classification that was also based on the example-based approach. Rios and Kavuluru [16] proposed a complete solution to the code assignment task addressed as the multi-label text classification problem that includes feature selection, training data selection, and probabilistic threshold optimization. Farkas and Szarvas [17] presented an interesting approach that acquires new rules and synonyms through the use of decision trees. In addition, support vector machines [18–20], *k*-nearest neighbors [21], Bayesian ridge regression [22], and matrix factorization [23] have also been adopted in previous research.

A number of studies exist concerning automated code assignment to English medical text, while the work reported in this paper focuses on encoding the physicians’ diagnoses written in Chinese. The words in Chinese sentences are not separated by any delimiters [24] and have no apparent morphological marker [25]. Moreover, a lot of Chinese words share the same or very similar lexical meaning. Given the differences in the linguistic features of English and Chinese, the approaches for English text cannot be applied directly to Chinese text. Furthermore, these features make semantic analysis indispensable in research associated with the processing of Chinese text. Mihalcea et al. [26] proposed an effective framework to measure the semantic similarity of text based on word-to-word similarity. In terms of semantic similarity between words or terms, some studies utilized the taxonomical information derived from existing knowledge resources [27–29], and others utilized the distributional statistics of electronic text [30–33]. With regard to the semantic similarity estimation of Chinese words, a classic method by Liu and Li [34] has been proposed on the basis of HowNet, a Chinese semantic knowledge base. Several subsequent studies [35, 36] were based on this method. Apart from these domain-independent approaches, a few studies have investigated semantic similarity estimation in the biomedical domain in recent years. Pedersen et al. [37] adapted six existing measures to the biomedical domain, where SNOMED-CT was used as the knowledge base and medical corpora in Mayo Clinic were used for distributional semantics implementation. Sánchez and Batet [38] extended their work by approximating concept semantics in terms of Information Content. McInnes and Pedersen [39] discovered the complementary effect between different measures in evaluating semantic similarity and relatedness. Among all the measures applied in these biomedical works, the context vector method by Patwardhan and Pedersen [33] was the only distributional approach and demonstrated promising performance. However, no study has been reported on Chinese semantic similarity calculation in the biomedical domain.

The current study focuses on ICD-10 code assignment to Chinese diagnoses because the coding process in China is primarily based on the diagnostic statements from the problem list in EMR. The results of this study can provide several insights into how Chinese medical text can be encoded effectively and how semantic similarity estimation between Chinese biomedical terms can be implemented.

## Methods

### Example-based model

Given the lack of mature tools and a complete knowledge base for Chinese medical language processing, automated code assignment to Chinese diagnoses cannot be fulfilled through the MLP approach. Based on the fact that encoding a diagnosis essentially refers to the selection of an ICD-10 code that can best describe the diagnosis, we proposed an example-based model that performs encoding by searching for the ICD-10 code with corresponding instances most similar to the given diagnosis. The efficacy of the example-based model in the automated encoding of diagnoses has been demonstrated by Pakhomov et al. [14], who based their study on the historical coding records of the Mayo Clinic. Unlike that, the present study is based on the standard diagnostic library (SDL).

SDL is a controlled medical terminology in the Chinese language that focuses on diagnostic phrases. It was drafted with WHO Collaborating Center for the Family of International Classifications as the leading organization, and published and popularized by the Statistical Information Center of the National Health and Family Planning Commission (NHFPC) of the People’s Republic of China^1^. This terminology comprises the standardized names of over 22,000 common diagnoses. Each diagnosis is assigned with a 6-digit ICD-10-CM code. Code assignment of these standard diagnoses is consistent with ICD-10 taxonomy, i.e., each diagnosis with a 6-digit code is a subdivision of the disease with the corresponding 4-digit code in ICD-10. An example is shown in Table 1. The 4-digit code A01.0 subsumes three diagnoses in SDL, namely “伤寒” (Typhoid), “伤寒性肝炎” (Typhoid hepatitis), and “伤寒性脑膜炎” (Typhoid meningitis). Each 4-digit ICD-10 code includes 2.28 diagnoses in SDL on the average. The publication and spread of SDL conducted by NHFPC aims at providing a guideline for diagnostic writing and code assigning to nationwide hospitals. Considering its good coverage on the diagnosis domain, SDL can serve as a reliable data set for our example-based model.

**Table 1:** An example of the standard diagnostic library

Standard diagnosis	English translation	Code
古典生物型霍乱	Classical biotype cholera	A00.000
埃尔托型霍乱	El Tor cholera	A00.100
霍乱	Cholera	A00.900
伤寒	Typhoid	A01.000
伤寒性肝炎	Typhoid hepatitis	A01.001
伤寒性脑膜炎	Typhoid meningitis	A01.002
副伤寒甲	Paratyphoid A	A01.100

In this study, the proposed method is based on SDL rather than on historical coding records from a medical institution for two main reasons. First, unlike SDL that is published by a government organization, historical coding records are relatively error-prone. Second, given that SDL is publicly available, the proposed example-based method is reproducible. And data sharing and experiment reproducibility are among the critical factors that can advance the research on data-driven informatics [18].

Our basic model, which is referred to herein as flat method, determines the subcategory code to a given diagnosis in the following manner:

$$ \underset{c\in {C}_{subcat}}{code= \arg \max sim\left(D,{T}_c\right),} $$

where *D* is the given diagnosis and *T*_*c*_ is denoted as the text segment derived by concatenating all diagnoses in SDL whose ICD-10-CM code is included in code *c* [e.g., *T*_*A*01.0_ = *T*_*A*01.000_ + *T*_*A*01.001_ + *T*_*A*01.002_ = “伤寒(Typhoid) 伤寒性肝炎(Typhoid hepatitis) 伤寒性脑膜炎(Typhoid meningitis)”]. In a way, *T*_*c*_ can be regarded as the textual description for code *c*. Besides, *C*_*subcat*_ is the set of all subcategory codes in ICD-10 and *sim* is the semantic similarity metric between two texts. Word segmentation is applied on text through ICTCLAS [40], an integrated Chinese lexical analyzer based on hierarchical hidden Markov model, with precision and recall over 90 %.

### Hierarchical method

**Fig. 1:**
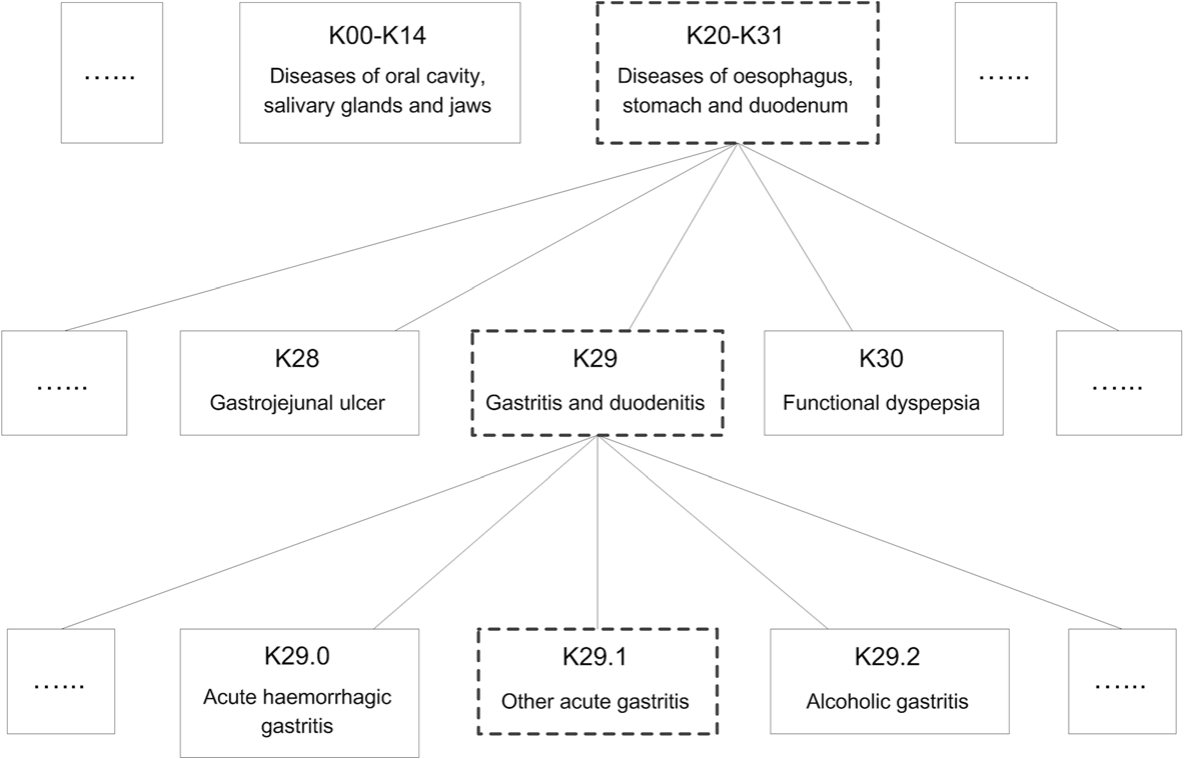
Example of the hierarchical coding method. Below the code is the description from the Tabular List of ICD-10, not the record from SDL

It should be noted that there’re more than 10,000 unique ICD-10 codes at the subcategory level (i.e., |*C*_*subcat*_| >10,000). Therefore, performing similarity calculation for the entire set of subcategory codes is time-consuming. By considering the hierarchical structure of ICD-10 codes where a child code represents a subdivision of its parent, we propose a hierarchy-based method that hierarchically determines the most suitable code until the subcategory code is obtained. To be specific, given a diagnosis *D*, we first determine the section code *c*_*sec*_ in the following manner:

$$ {c}_{sec}=\underset{c\in {C}_{sec tion}}{ \arg \max }sim\left(D,{T}_c\right), $$

where *C*_*section*_ is the set of all section codes in ICD-10. Then we select *c*_*cat*_ from the category codes under *c*_*sec*_ where ${T}_{c_{cat}}$ has the highest semantic similarity with *D*. Finally, among all the subcategory codes that are the subdivision of *c*_*cat*_, the most suitable one is determined. The section code is determined first because a code at a higher level is too generic. An example illustrating the hierarchical coding process is shown in Fig. 1. Given a diagnostic statement *D* = “急性胃炎” (acute gastritis), *T*_*K*20 − *K*31_ is found to have the highest semantic similarity to *D*; thus, K20-K31 is determined as the most appropriate section code. In the same manner, category code K29 is determined, and subcategory code K29.1 is eventually obtained.

Compared with the flat method that searches within over 10,000 subcategory codes, the hierarchical method only examines the entire section codes (262), the category codes under a specific section code (less than 40), and the subcategory codes under a specific category code (at most 10). Therefore, the number of codes that need to be examined is greatly reduced. This feature can improve the efficiency of the example-based code assignment.

### Measure of text semantic similarity

The proposed encoding method selects a 4-digit code that can best describe a particular diagnosis according to instances from SDL, so the measure of text semantic similarity is crucial to the performance of code assignment. Mihalcea et al. [26] proposed an effective approach that combines the metrics of word-to-word similarity and word specificity. This method was adopted and modified in the current study to measure the semantic similarity of text.

According to [26], the semantic similarity between two text segments *T*_1_ and *T*_2_ is determined by the formula:

$$ sim\left({T}_1,{T}_2\right)=\frac{1}{2}\left(\frac{{\displaystyle \sum_{w\in \left\{{T}_1\right\}}\left( \max Sim\left(w,{T}_2\right)\cdot idf(w)\right)}}{{\displaystyle \sum_{w\in \left\{{T}_1\right\}}idf(w)}}+\frac{{\displaystyle \sum_{w\in \left\{{T}_2\right\}}\left( \max Sim\left(w,{T}_1\right)\cdot idf(w)\right)}}{{\displaystyle \sum_{w\in \left\{{T}_2\right\}}idf(w)}}\right), $$

where *maxSim*(*w*,*T*) is the highest word-to-word similarity between word *w* and any word from *T*, and *idf*(*w*) is the inverse document frequency of *w*, which is defined as the total number of documents in a corpus divided by the number of documents that contain word *w*. This metric demonstrates an effective performance in the estimation of text semantic similarity.

However, a problem will occur if this metric is adopted directly. All words in *T*_1_ will contribute to the similarity calculation no matter how low the similarity of the word to *T*_2_ [*maxSim*(*w*,*T*_2_)] is; this condition is unreasonable. Given *T*_1_ = “男性盆腔炎” (male pelvic inflammatory disease) and *T*_2_ = “女性盆腔炎” (female pelvic inflammatory disease), similarity between *T*_1_ and *T*_2_ should have resulted entirely from “盆腔炎” (pelvic inflammatory disease) since “男性” (male) and “女性” (female) are irrelevant. However, “男性” (male) can still contribute a similarity of *maxSim*(“男性 (male)”, *T*_2_) = *sim*(“男性 (male)”, “女性 (female)”) = 0.23 because the two words, “男性” (male) and “女性” (female), have a semantic similarity of 0.23 according to the next subsection (HowNet-based). It is illogical to including irrelevant words in the similarity measure even if they have a low but positive word similarity. To overcome this problem, we set a threshold *θ* for the word-to-word similarity and obtain the following similarity metric:

$$ sim\left({T}_1,{T}_2\right)=\frac{1}{2}\left(\frac{{\displaystyle \sum_{w\in S\left({T}_1,{T}_2,\theta \right)}\left( \max Sim\left(w,{T}_2\right)\cdot idf(w)\right)}}{{\displaystyle \sum_{w\in \left\{{T}_1\right\}}idf(w)}}+\frac{{\displaystyle \sum_{w\in \left({T}_2,{T}_1,\theta \right)}\left( \max Sim\left(w,{T}_1\right)\cdot idf(w)\right)}}{{\displaystyle \sum_{w\in \left\{{T}_2\right\}}idf(w)}}\right), $$

where *S*(*T*_1_, *T*_2_, *θ*) = {*w* ∈ {*T*_1_}|*maxSim*(*w*, *T*_2_) ≥ *θ*} and similarly for *S*(*T*_2_, *T*_1_, *θ*). By introducing the similarity threshold, the irrelevant words that have a positive but low similarity are not considered, thus increasing the accuracy of text semantic similarity estimation in code assignment.

The metric of word-to-word similarity is introduced in the following subsection. Inverse document frequency is calculated based on the discharge summaries of our partner hospital in Beijing.

### Semantic similarity of words

As stated before, semantic similarity between words or terms can be estimated through knowledge-based methods and corpus-based methods. In the biomedical domain, SNOMED-CT and UMLS were generally used as the knowledge base and medical corpora were used for distributional semantics implementation. However, SNOMED-CT or UMLS does not have a Chinese version, and there does not exist a medical knowledge base as complete as SNOMED-CT or UMLS in China. Therefore, we chose HowNet, a Chinese domain-independent knowledge base, to implement the knowledge-based method. We also employed distributional approach for semantic similarity estimation.

#### Similarity estimation based on HowNet

Launched in 1999, HowNet is a knowledge base system that describes the concepts represented by Chinese words and reveals the relationship among these concepts [36]. Similar to WordNet, knowledge in HowNet is organized in a hierarchical tree-like structure. The difference between them is that sememe is considered the smallest and most basic semantic unit in HowNet, while concept is the smallest unit in WordNet. In HowNet, a Chinese word is represented by a set of concepts, and a concept is described by a group of sememes. So the semantic similarity between words can be calculated based on this knowledge base.

The semantic similarity metric developed by Liu and Li [34], in which similarity between words is defined as the maximum of the similarities between the concepts of the two words, is adopted in the present study. Similarity between concepts was measured by the similarities between the corresponding sememes. The similarity of sememes *s*_1_ and *s*_2_ is calculated based on the shortest distance between these two sememes in HowNet:

$$ sim\left({s}_1{s}_2\right)=\frac{\alpha }{distance\left({s}_1{s}_2\right)+\alpha }, $$

where $\alpha$ is an adjustable parameter. The recommended values in [34] were used for the parameters in this study.

However, this approach will fail occasionally if a word is vocational and not included in HowNet [e.g., “木糖葡萄球菌” (staphylococcus xylosus)] because words that comprise diagnostic statements generally belong to the biomedical domain whereas HowNet is a domain-independent knowledge base. Here, the string similarity of words is applied when the semantic similarity estimation fails:

$$ sim\left({w}_1,{w}_2\right)=\frac{len\left(LCS\left({w}_1,{w}_2\right)\right)}{len\left({w}_1\right)+len\left({w}_2\right)-len\left(LCS\left({w}_1,{w}_2\right)\right)}, $$

where *len*(*w*) is the length of the string or sequence *w* and *LCS*(*w*_1_, *w*_2_) is the longest common subsequence between words *w*_1_ and *w*_2_.

#### Similarity estimation based on distributional semantics

In contrast with knowledge-based methods that represent semantics in a taxonomical knowledge base or ontology, distributional methods determine the semantics of terms from the way in which these terms are distributed across a large body of domain-relevant text [41]. These methods are based on the hypothesis that words are similar if their contexts are similar [42]. The context vector method proposed by Patwardhan and Pedersen [33] has been applied the most often in the biomedical domain. In this method, a co-occurrence matrix is constructed from a corpus of textual documents. Each row in the matrix can be viewed as a *word vector*, whose dimensions are content words from the corpus (each dimension corresponds to one content word). Each cell in the word vector of word *w* is the frequency that the content word co-occurs with *w* within a specified window of context. The word vector provides an evident representation of word semantics through the contextual co-occurrence information. Accordingly, the semantic similarity of two words *w*_1_ and *w*_2_ can be measured as the cosine of the angle between their word vectors:

$$ sim\left({w}_1{w}_2\right)=\frac{{\overset{\rightharpoonup }{v}}_1\cdot {\overset{\rightharpoonup }{v}}_2}{\left|{\overset{\rightharpoonup }{v}}_1\right|\left|{\overset{\rightharpoonup }{v}}_2\right|}, $$

where ${\overset{\rightharpoonup }{v}}_1$ and ${\overset{\rightharpoonup }{v}}_2$ are the word vectors for *w*_1_ and *w*_2_, respectively. This method was adopted in the current study to calculate the semantic similarity between words.

However, one issue will arise if we apply this method in the Chinese language. As stated in the Background section, there are no delimiters between words in Chinese text. A text segment in Chinese is actually a string of Chinese characters, and word segmentation is required to separate the words. Since word segmentation tools cannot possibly achieve 100 % accuracy, word vectors may sometimes fail to represent the correct distributional semantics. An alternative was proposed in this study that the contextual semantics of a word is not represented by content words but by content characters (i.e., the dimensions of a word vector are no longer Chinese words but Chinese characters). This new way of constructing word vectors makes sense because unlike English, Chinese characters do to some extent represent semantics. For example, in the word “苹果” (apple), the English character “a” or “p” does not provide any semantics to “apple”, while the Chinese character “果” does provide some semantics to “苹果” (apple) because it has the meaning of “fruit”. A similar approach in the English context is Schutze’s Wordspace model [31] that represents distributional semantics based on four-grams. Therefore, apart from constructing word vectors according to the original method (henceforth word-based vector), we also constructed the vectors based on Chinese characters (henceforth char-based vector).

To implement the distributional approaches, we collected 54,136 inpatient discharge summaries from 2012 to 2014 at our partner hospital in Beijing. These documents, which have been de-identified, were used as the corpus for the construction of word vectors because they have better coverage of words in the biomedical domain. Pre-processing was performed on the corpus text including the removal of stop words, numerals, and punctuation. Word segmentation was performed through ICTCLAS. For the word-based vector method, the window size was set as three content words around the word. And for the char-based vector method, the window size was set as seven content characters around the word.

### Evaluation of proposed methods

To evaluate the validity of the proposed encoding methods, we collected 16,330 manually coded instances extracted from the EMRs of our partner hospital as the test set. Each coding instance consists of a diagnostic statement and the corresponding ICD code. Code assignment of these instances was conducted by professional coders. With regard to the role of medical coding, it has been reported that manual code assignment in the US is primarily for the purpose of billing and is believed to inadequately reflect the clinical reality. However, to the best of our knowledge, the primary purpose of manual coding in Chinese hospitals is the direct translation from clinical statements to clinical codes for reporting to the Health and Family Planning Commissions. Clinical decision support and billing only serve as the secondary purposes. Accordingly, an appropriate match exists between the diagnoses and the manually assigned codes in the test set, which is valid for the evaluation of the proposed encoding methods.

Micro-averaged precision, recall, and F-score were used in this study as the evaluation metrics. Precision and recall are defined as follows:

$$ Precision=\frac{{\displaystyle {\sum}_ct{p}_c}}{{\displaystyle {\sum}_ct{p}_c}+{\displaystyle {\sum}_cf{p}_c}}, $$

$$ Recall=\frac{{\displaystyle {\sum}_ct{p}_c}}{{\displaystyle {\sum}_ct{p}_c}+{\displaystyle {\sum}_cf{n}_c}}, $$

where *tp*_*c*_, *fp*_*c*_, and *fn*_*c*_ represent true positives (correctly assigned instances), false positives (incorrect assignments by automated methods), and false negatives (correct instances omitted by automated methods), respectively, of code *c*. And F-score is the harmonic mean of precision and recall with equal weights according to the following formula:

$$ Micro{F}_1=\frac{2\cdot Precision\cdot Recall}{Precision+ Recall}. $$

The proposed methods were applied on the test set and the performance is shown and discussed in the next section.

## Results and discussion

As mentioned before, code assignment is usually performed at the 4-digit level. So we focus on the accuracy of our proposed encoding methods at 4-digit level. Nonetheless, in order to provide a comprehensive performance demonstration, we display the results at both 4-digit and 3-digit level. At 4-digit level, code assignment is correct only when two codes are equal in the first 4 digits; while at 3-digit level, code assignment is considered correct when two codes are equal in the first 3 digits (e.g., A01.0 and A01.1).

**Fig. 2:**
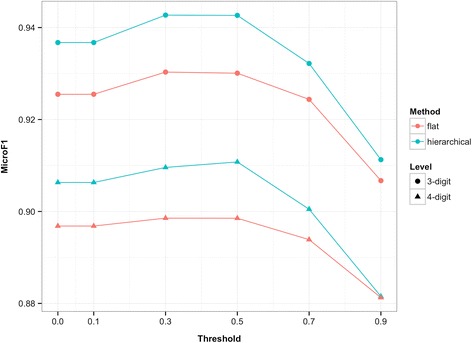
Performance with semantic similarity estimation through HowNet-based method

**Fig. 3:**
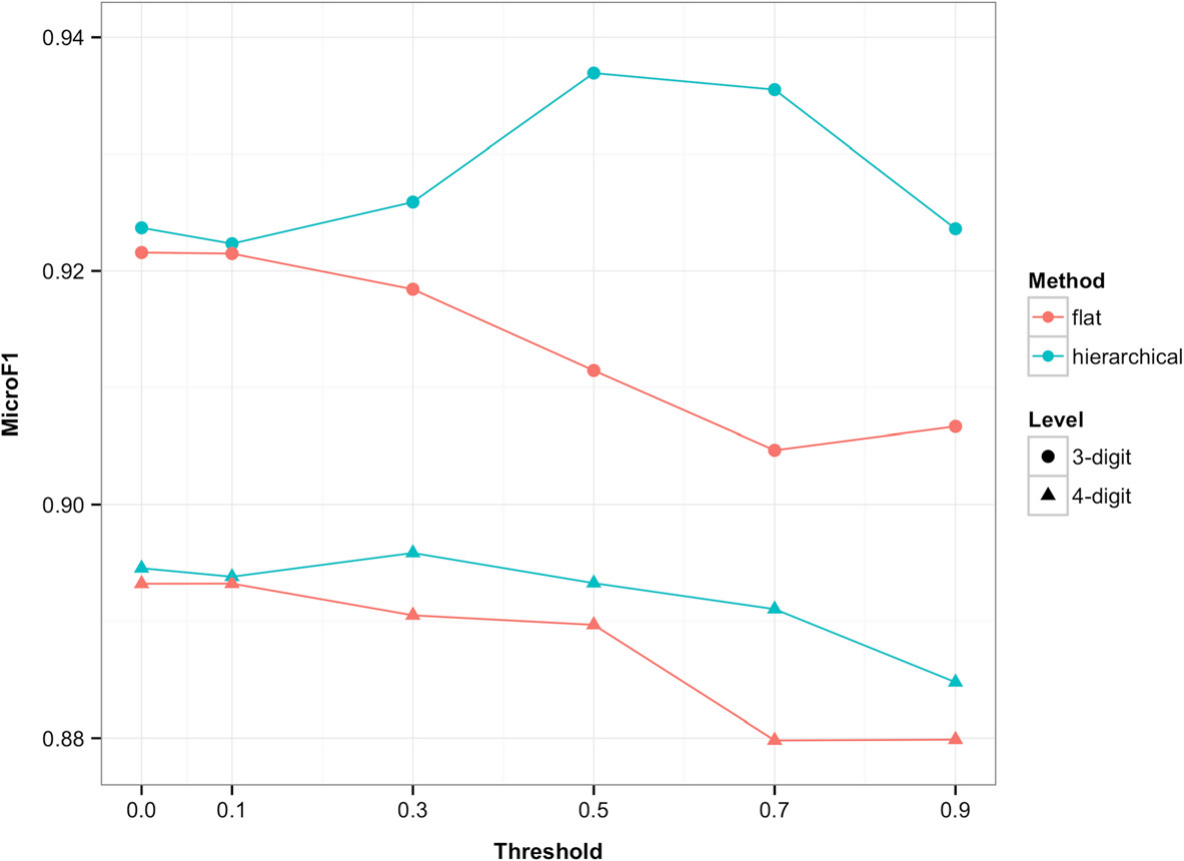
Performance with semantic similarity estimation through word-based vector method

**Fig. 4:**
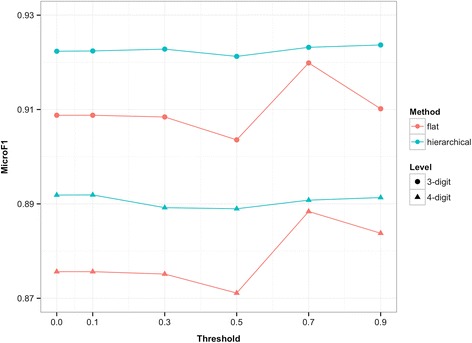
Performance with semantic similarity estimation through char-based vector method

In the present study, two types of example-based methods were developed, namely, flat method and hierarchical method. One knowledge-based and two distributional approaches were applied for word-to-word semantic similarity estimation. And word similarity threshold were also introduced. Results under these settings are shown from Figs. 2, 3 and 4. For the purpose of direct illustration, we only display the coding performance by F-score. Detailed results (with precision and recall) are shown in Additional file 1.

### Word similarity threshold

Under any semantic similarity measure, the 4-digit coding accuracy of both flat and hierarchical methods can be improved with an appropriate threshold setting. Specifically, under the HowNet-based measure, flat method reaches its optimum with a threshold value of 0.3 and hierarchical method is optimal with 0.5; under the word-based vector measure, the optimal threshold setting for flat method is 0.1 and for hierarchical method it is 0.3; under the char-based vector measure, the optimal value is 0.7 for flat method and 0.1 for hierarchical method. It should be noted that the threshold setting of 0 is equal to no introduction of the word similarity threshold, so the validity of this introduction is confirmed. However, a very high threshold may decrease the accuracy of the proposed methods. This tendency is similar at 3-digit level.

With an appropriate threshold setting, we can filter out the irrelevant words with low but positive similarity that may interfere in the calculation of text similarity. This will enhance the accuracy of semantic similarity estimation and contribute to the code assignment. However, if the threshold is set too high, some words that should’ve been taken into account will be filtered out [e.g., “鼻” (nose) and “鼻炎” (rhinitis), with a similarity of 0.61 (under word-based vector measure)]. This situation will result in information loss during semantic similarity estimation and will negatively influence the coding accuracy.

### Effectiveness of the hierarchical method

In addition to the validity of introducing word similarity threshold, the results also reveal that hierarchical method outperforms flat method in terms of coding accuracy at both 4-digit and 3-digit level. This result makes sense because hierarchical method additionally takes advantage of the hierarchical nature of the ICD-10 codes. Compared with the flat method that searches within the entire set of subcategory codes, the hierarchical method guides the code assignment process by narrowing the search scope step by step until the suitable subcategory code is identified. Such a process can prevent code assignment from being misled by the wrong codes that coincidentally possess descriptions that are very similar to the given diagnosis. We take the diagnosis *D* = “耳神经痛” (auricular neuralgia) that should have been classified into H92.0 as an example. In SDL, the corresponding description *T*_*H*92.0_ is “耳痛” (earache), but the flat method will select G58.0 instead of H92.0 because G58.0 happens to have the description “肋间神经痛” (intercostal neuralgia), which is more similar to the diagnostic text *D* [i.e., *sim*(*D*, *T*_*H*92.0_) < *sim*(*D*, *T*_*G*58.0_)]. The hierarchical method overcomes this problem by determining section code H90-H95 in the first place instead of G50-G59 because *sim*(*D*, *T*_*H*90 − *H*95_) > *sim*(*D*, *T*_*G*50 − *G*59_). This is natural since diagnoses with codes under H90-H95 are certainly more relevant to the diagnosis that belongs to H92.0, thus overcoming the coincidence. Then category code H92 is determined, and H92.0 is eventually selected.

Moreover, one can find from the figures that the trends of curves at 4-digit level are consistent with those at 3-digit level (less consistent in Fig. 3, but the relationship between flat and hierarchical methods still holds). Therefore, it can be inferred that the superior performance of the hierarchical method in 4-digit accuracy is largely the result of its superior performance in 3-digit accuracy.

Apart from coding accuracy, time complexity, which was the motivation for the introduction of the hierarchical method, is also investigated. Table 2 shows the average running times required for a diagnosis to be encoded with the two methods under different word similarity measures. Apparently, the hierarchical method is much faster than flat method.

**Table 2:** Average running times to encode a diagnosis

Measure	Flat method	Hierarchical method
HowNet-based	3.15 s	1.24 s
Word-based vector	3.02 s	1.16 s
Char-based vector	3.68 s	1.34 s

Consequently, hierarchical method is effective in automated code assignment with both high accuracy and short running time. So it is demonstrated that the hierarchical nature of ICD-10 codes can greatly enhance the performance of automated code assignment to Chinese diagnoses.

### Comparison of semantic similarity measures

In order to compare the performance of the knowledge-based method and two distributional methods for semantic similarity estimation, we summarize the optimal F-scores obtained under two encoding methods and three similarity measures, which are shown in the first three rows in Table 3.

**Table 3:** F-scores under the optimal word similarity threshold

Measure	4-digit		3-digit	
	Flat	Hierarchical	Flat	Hierarchical
HowNet-based	0.8986	0.9108	0.9303	0.9427
Word-based vector	0.8932	0.8958	0.9216	0.9369
Char-based vector	0.8884	0.8919	0.9199	0.9237
Exact matching	0.7933	0.7928	0.8532	0.8564

The results reveal that in this study, measuring semantic similarity between Chinese medical words using HowNet, a domain-independent knowledge base, achieves higher coding accuracy than using distributional approaches. It demonstrates the effectiveness and applicability of the HowNet-based measure (combined with string similarity metric) in evaluating semantic similarity in a specific domain. However, the HowNet-based method does not outperform the distributional methods much; the performance is close. According to Pedersen et al. [37], the quality of semantic similarity estimation using the context vector method is heavily dependent on corpus size. A corpus with small size may bias the distributional statistics and influence the accuracy of distributional semantics implementation. In their work, a sharp increase in performance was observed when corpus size was raised from 100,000 to 300,000. In the current study, we could only collect 54,136 discharge summaries from our partner hospital, due to the incomplete information systems and imperfect data storage in Chinese hospitals. Therefore, with a larger medical corpus, it is very likely that the distributional approaches will achieve superior performance over the knowledge-based approach in automated diagnoses encoding. Another issue worth considering is the finding in [37] that compared with distributional measures, semantic similarity estimation using knowledge-based measures tended to better align with coders than with physicians. And code assignment in the test set we applied was conducted by coders. This may also explain why the HowNet-based method outperformed the context vector method in our work.

An interesting finding from Table 3 is that constructing word vectors based on Chinese characters can obtain close performance as word-based method. Compared with the original method (word-based) proposed under the English language, representing distributional semantics using content characters only has slight influence on coding accuracy but will save the effort of word segmentation. Accordingly, the char-based method demonstrates an acceptable new way to represent contextual semantics in the Chinese language.

The example-based method proposed in this study is based on semantic similarity estimation because of the distinct features of the Chinese language described before. Hence, we are interested in whether semantic analysis is indeed indispensable in dealing with Chinese medical text. An alternative measure called exacting matching, under which the similarity between two words is 1 only if they are equal in string and 0 otherwise, was suggested. The fourth row in Table 3 shows the coding accuracy when we apply this word similarity measure to the encoding methods. The result reveals a sharp decrease in encoding performance without semantic similarity measures. Therefore, we have demonstrated that exact matching, which is usually applied to match the words in English text, is ineffective in dealing with Chinese text. Consequently, semantic analysis plays a crucial role in automated code assignment to Chinese diagnoses and is proved indispensable in research focusing on Chinese medical text.

The efficacy of semantic similarity estimation can also be revealed through specific examples of code assignment by our proposed methods. Given a diagnosis “慢性(chronic) 肾功能(renal) 不全(insufficiency)” that should be encoded into N18.9, the description from SDL selected by our method is “慢性(chronic) 肾功能(renal) 衰竭(failure)” with the corresponding code N18.9. This assignment is reasonable since chronic renal insufficiency is likely to be resulted from chronic renal failure. For the diagnosis “三尖瓣(tricuspid) 闭锁(atresia) 不全(insufficiency)”, our method detects that “闭锁” (atresia) is semantically similar to “关闭” (closure) and determines the correct match “风湿性(rheumatic) 三尖瓣(tricuspid) 关闭(closure) 不全(insufficiency)” from SDL.

### Performance discussion

The proposed methods can achieve the optimum F-score of 91.08 % through hierarchical encoding method and HowNet-based semantic similarity measure. Another issue worth considering is the comparison with other automated clinical coding systems on the coding performance. According to our knowledge and the systematic review by Stanfill et al. [43], the performance of existing research on automated code assignment varied widely. And the variety in the evaluation metrics applied (e.g., precision, recall, specificity, and accuracy) made the performance values difficult to compare and contrast. Nevertheless, if we only focus on studies with precision and recall as the evaluation metrics, the performance ranges from less than 10 % to nearly 100 % in terms of precision and ranges from over 20 % to nearly 100 % in terms of recall. Based on the performance statistics in [43], our method outperforms the majority of existing automated coding systems in terms of precision and recall. Apart from evaluation metrics, variety between coding tasks also exists in document types and classification schemes. Compared with those concerning code assignment to discharge summaries or radiology reports, our study only focuses on diagnoses encoding, which makes the task less difficult. However, since ICD-10 contains a larger set of possible codes (over 10,000 at the 4-digit level) than most other classification schemes like ICD-9 and ICD-8, the difficulty of accurate code selection is increased.

### Application of the method

**Fig. 5:**
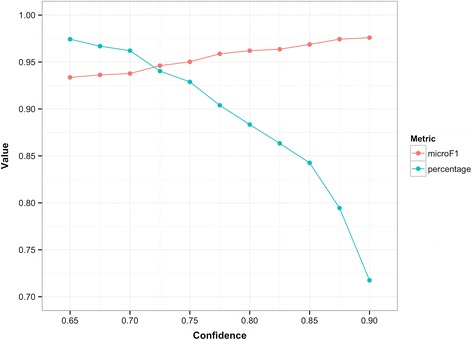
Coding accuracy and percentage of diagnoses with confidence above the given threshold

Given that our encoding method cannot possibly achieve 100 % accuracy in assigning the correct codes to diagnoses, this method must be able to detect incorrect code assignment during practical application. A measure of confidence, which is defined as *sim*(*D*, *T*_*c* *_), where *D* is the diagnostic text and c* is the code that is eventually assigned to *D*, is introduced. Confidence can represent the extent to which the selected code can describe the given diagnosis with instances from SDL and can reflect the reliability of code assignment. The Wilcoxon rank-sum test reveals a significant difference between the confidences of the correctly and incorrectly encoded diagnoses in the test set. This result shows that confidence can serve as an effective metric to detect incorrect outputs. Figure 5 shows the coding accuracy and percentage of diagnoses with confidence values that exceed the threshold.

**Fig. 6:**
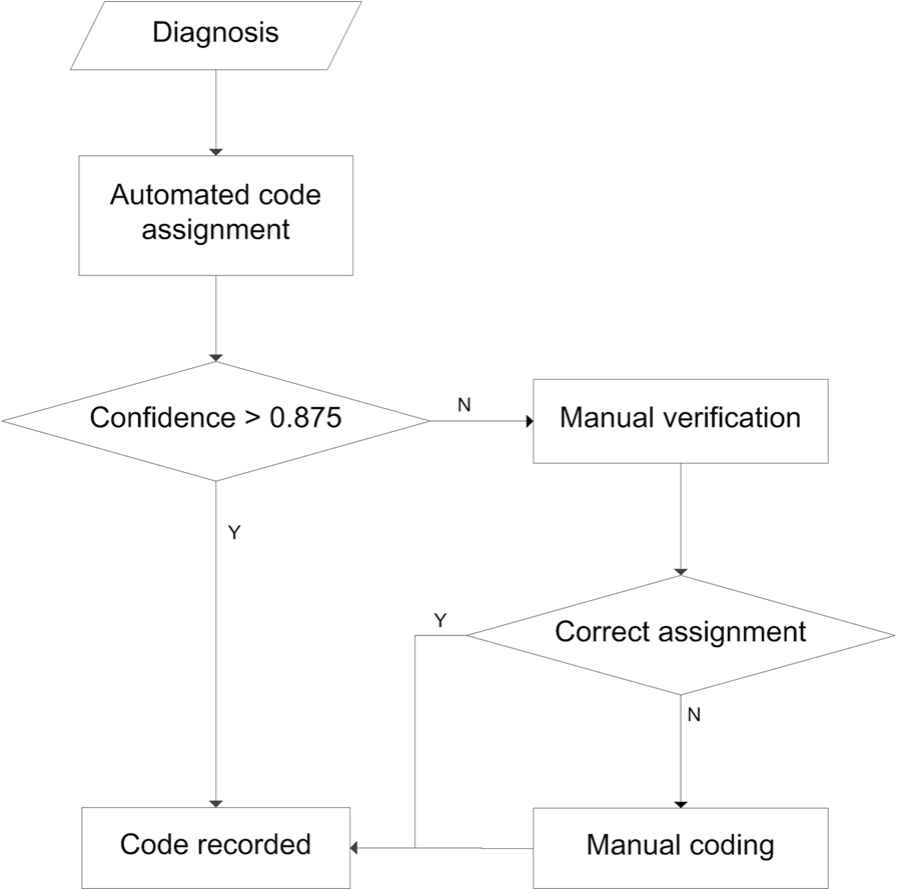
Coding process of the proposed method in practical application

When the confidence setting is high, the number of diagnoses with confidence values that exceed the threshold decreases, whereas the coding accuracy for such diagnoses is enhanced. Under a confidence setting of 0.875, the proposed method can automatically encode 79.44 % of the diagnoses with an F-score of 97.43 %. Given the very high coding accuracy, the codes that are assigned to this part of the diagnoses can be directly produced without manual verification. The remaining 20.56 % of the diagnoses are coded with an F-score of 69.11 %. The coding results for these diagnoses can only serve as reference and must be verified or recoded by medical coders. Then the coding process of the proposed method in practical application is presented in Fig. 6.

Given a diagnostic statement, a code and a corresponding confidence value are assigned automatically through the proposed method. The code will be directly recorded if the confidence exceeds 0.875. Otherwise, the coding result will be submitted to medical coders for verification or recoding. Nearly four-fifth of all diagnoses can be encoded automatically through our proposed method with a high F-score of 97.43 %, and only one-fifth needs to be submitted for manual verification. This coding process will greatly reduce the workload of medical coders and improve the efficiency of medical code assignment in hospitals.

### Limitations and future work

Given the lack of complete knowledge resources for natural language processing of Chinese medical text, we have developed an effective example-based method to automatically assign ICD-10 codes to Chinese diagnoses by applying word segmentation and semantic similarity estimation, and exploiting the hierarchical nature of the ICD-10 codes. However, room for improvement still exists. Because of the lack of a complete medical knowledge base in Chinese, we could only implement the knowledge-based semantic similarity measure through a domain-independent one. And the distributional measures were implemented with a medical corpus of relatively small size. Moreover, ICTCLAS may incorrectly segment words. Such limitations negatively affect the performance of the proposed example-based method. With the increasing number of studies on the construction of a complete and publicly available Chinese medical knowledge base and the continuous upgrade of hospital information systems in China, a comprehensive study on the semantic similarity estimation between Chinese words in the biomedical domain could be conducted and would contribute greatly to the performance of the automated code assignment.

## Conclusion

In this paper, an example-based method for the automatic assignment of ICD-10 codes to diagnoses written in Chinese was developed. The hierarchical nature of ICD-10 codes was maximized, and effective coding performance was achieved. Semantic similarity estimation between Chinese words was successfully implemented for the first time in the biomedical domain with both knowledge-based and distributional approaches, with consideration of characteristics of the Chinese language. The indispensability of semantic similarity estimation in assigning codes to Chinese medical text was also demonstrated. F-score of 91.08 % was obtained for all diagnoses in the test set. In the coding process designed for practical application, nearly four-fifth of the diagnoses can be coded automatically with the proposed method, with high F-score of 97.43 % and no need for manual verification. The proposed method can support and enhance the efficiency of the code assignment process in Chinese hospitals. Construction of a complete Chinese medical knowledge base may lead to additional improvements in the performance of the proposed automated encoding method.

## Additional file

Additional file 1: Coding performance under all settings. The detailed precision, recall, and F-score of the proposed algorithm under two encoding methods, three semantic similarity measures, and various word similarity thresholds. (XLSX 53 kb)

## Abbreviations

EMR: electronic medical record
ICD: International Classification of Diseases
MLP: medical language processing
NHFPC: National Health and Family Planning Commission
SDL: standard diagnostic library
WHO: World Health Organization

## Footnotes

1 Although having being popularized nationwide since 2013, SDL as a national standard is still under application. As a result, SDL is publicly available among medical institutions in China, but not available on the Internet. The only available reference is the project number of SDL, which is GB/T14396-2012.
